# Beware of pharyngeal *Fusobacterium nucleatum* in COVID-19

**DOI:** 10.1186/s12866-021-02336-6

**Published:** 2021-10-11

**Authors:** Lirong Bao, Cheng Zhang, Jinglu Lyu, Caixia Yan, Ranran Cao, Ming Pan, Yan Li

**Affiliations:** 1 State Key Laboratory of Oral Diseases, National Clinical Research Center for Oral Diseases, West China Hospital of Stomatology, Sichuan University, Chengdu, 610041 China; 2 Sichuan Center for Disease Control and Prevention, Chengdu, 610041 China

**Keywords:** SARS-CoV-2, COVID-19, *Fusobacterium nucleatum*, *Faecalibacterium prausnitzii*, Metagenomic next-generation sequencing

## Abstract

**Background:**

*Fusobacterium nucleatum* (*F. n*) is an important opportunistic pathogen causing oral and gastrointestinal disease. *Faecalibacterium prausnitzii* (*F. p*) is a next-generation probiotic and could serve as a biomarker of gut eubiosis/dysbiosis to some extent. Alterations in the human oral and gut microbiomes are associated with viral respiratory infection. The aim of this study was to characterise the oral and fecal bacterial biomarker (i.e., *F. n* and *F. p*) in COVID-19 patients by qPCR and investigate the pharyngeal microbiome of COVID-19 patients through metagenomic next-generation sequencing (mNGS).

**Results:**

Pharyngeal *F. n* was significantly increased in COVID-19 patients, and it was higher in male than female patients. Increased abundance of pharyngeal *F. n* was associated with a higher risk of a positive SARS-CoV-2 test (adjusted OR = 1.32, 95% CI = 1.06 ~ 1.65, *P* < 0.05). A classifier to distinguish COVID-19 patients from the healthy controls based on the pharyngeal *F. n* was constructed and achieved an area under the curve (AUC) of 0.843 (95% CI = 0.688 ~ 0.940, *P* < 0.001). However, the level of fecal *F. n* and fecal *F. p* remained unaltered between groups. Besides, mNGS showed that the pharyngeal swabs of COVID-19 patients were dominated by opportunistic pathogens.

**Conclusions:**

Pharyngeal but not fecal *F. n* was significantly increased in COVID-19 patients, clinicians should pay careful attention to potential coinfection. Pharyngeal *F. n* may serve as a promising candidate indicator for COVID-19.

**Supplementary Information:**

The online version contains supplementary material available at 10.1186/s12866-021-02336-6.

## Introduction

Severe acute respiratory syndrome coronavirus 2 (SARS-CoV-2) is highly transmissible and pathogenic and has caused a pandemic coronavirus disease 2019 (COVID-19), which threatens human health and public safety. According to the World Health Organization (WHO) (https://covid19.who.int/), as of August 13, 2021, there have been more than 205 million confirmed cases of COVID-19 and more than 4.3 million deaths. COVID-19 is a complex multisystem disorder [1]. Common symptoms of COVID-19 include fever, fatigue and dry cough. Severe infections can lead to pneumonia, severe acute respiratory syndrome, kidney failure, and even death [2]. In addition to respiratory symptoms, COVID-19 can also experience oral/pharyngeal manifestations, such as sore throat, dry mouth, loss of taste, burning sensation and tongue enlargement [3, 4], and gastrointestinal symptoms like diarrhea, nausea, and vomiting [5, 6].

The pharynx is one of the main entrance into the gastrointestinal tract for microorganisms. Both the pharynx and gastrointestinal tract are recognised as crucial sites for the pathogenesis of SARS-CoV-2 infection [7, 8]. SARS-CoV-2 infection and invasion in these sites may cause local microbiome dysbiosis and thus lead to secondary bacterial infection [9–11]. The host-microbe interactions in the two sites are complex and key for the understanding of the physiology and mechanism of the immune response and microbiome during SARS-CoV-2 infection [8, 12, 13]. Each disease has its own specific microbial characteristics. Metagenomic next-generation sequencing (mNGS) analysis has revealed the pharyngeal microbiota alterations of SARS-COV-2 infected patients [8, 12, 14, 15], and oropharyngeal microbiota alterations were associated with COVID-19 severity [12]. A recent study [7] found that several oral microbial markers (like TM7, *Haemophilus*, *Actinomyces*, *Prevotella*, *Oribacterium* and *Fusobacterium*) could specifically identify patients with COVID-19 from the health controls in the random forest model.

*Fusobacterium nucleatum* (*F. n*), an anaerobic oral commensal associated with periodontitis, is usually found in respiratory and gastrointestinal tracts [16–19]. *F. n* is considered as a biomarker of chronic obstructive pulmonary disease patients’ lung function deterioration [20] and the development of inflammatory bowel disease and even colorectal cancer [21, 22]. Emerging evidence has demonstrated that the culture supernatant of *F. n* induced the up-regulation of ACE2 expression in human respiratory epithelial cells and the release of pro-inflammatory cytokines IL-6 and IL-8 [23] and thus *F. n* may play a synergistic role in the progression of SARS-CoV-2 infection [24]. *Faecalibacterium prausnitzii* (*F. p*) is an beneficial commensal anaerobe. Tang L et al. reported that the abundance of *F. p* significantly decreased in the critical COVID-19 patients compared with the general patients [25]. Interestingly, some microbes correlated inversely with SARS-CoV-2 loads in fecal samples from COVID-19 patients could downregulate the expression of ACE2 [26], suggesting that SARS-CoV-2 may inhibit microorganisms that are unfavorable for its infection. The concept of *F. n* and/or *F. p* serving as non-invasive diagnostic tools for specific diseases has been demonstrated in many studies [20, 27]. Act as the “harmful” and “beneficial” bacteria respectively [27], the potential roles of *F. n* and *F. p* in COVID-19 became an interesting future research topic.

In our study, we examined the carriage of *F. n* in pharyngeal swab samples of COVID-19 patients and healthy controls by quantitative real-time polymerase chain reaction (qPCR). We also investigated the relative abundance of *F. n* and *F. p* in the SARS-CoV-2 positive/negative fecal samples of COVID-19 patients. Besides, metagenomic next-generation sequencing (mNGS) was conducted in 10 randomly selected COVID-19 patients’ pharyngeal swabs to characterize the microbial composition and diversity, and then the correlation of the viral loads and the common bacteria were performed.

## Materials and methods

### Sample collection

A total of 64 laboratory-confirmed COVID-19 cases and 19 healthy controls were included in this study. All the participants are the local residents of Sichuan province, who share similar living environments and dietary habits. Eighty-three samples, including 38 pharyngeal swab samples (28 COVID-19 patients and 10 healthy controls) and 45 fecal samples (36 COVID-19 patients and 9 healthy controls) were collected. The samples were collected only once for each participant. Of the 36 fecal samples from patients, 26 were tested positive for SARS-CoV-2 and 10 were negative based on our previous study [28]. All samples were stored in sterile containers frozen at − 80 °C instantly after heat-inactivated [29]. The collection, transportation, storage and testing of samples were strictly managed and conducted in the biosafety level-2 (BSL-2) and biosafety level-2 enhanced (BSL-2 +) facilities of Sichuan Provincial Center for Disease Control and Prevention(CDC) with full personal protective equipment according to highly pathogenic microorganisms of type II according to the Protocol on Prevention and Control of COVID-19 (seventh edition) [30].

All the participants were diagnosed after laboratory confirmation and categorized into four disease severity, i.e., asymptomatic infection (individuals with positive detection by reverse transcriptase polymerase chain reaction and no symptoms), mild illness (patients with mild clinical symptoms and normal CT imaging), moderate illness (Patients with fever and mild respiratory symptoms, radiological findings of pneumonia, and normal range of vital signs), and severe illness (patients with at least one of the the following criteria: respiratory distress ≥30/min; oxygen saturation ≤ 93% in resting state; arterial partial pressure of oxygen [PaO_2_]/ fraction of inspired oxygen [FiO_2_] ≤300 mmHg), according to the guidelines of diagnosis and treatment of COVID-19 (trial version 7) [31].

### Nucleic acid extraction

All the samples were thawed at room temperature and pre-treated as follows. Two hundred milligram of feces were suspended in 2 mL TRIzol (Trizol@ Reagent, Invitrogen, USA), keep still for 10 min and then the supernatant was collected. The pharyngeal swab samples were oscillated on a shaker for 30 s, and the swab lotions were collected. Co-extraction of genomic DNA & total RNA from fecal and pharyngeal swab samples was carried out with NP968 Nucleic Acid Extraction System (Xi’an Tianlong Science & Technology Co., LTD, Xi’an, China). All samples were stored frozen at − 20 °C.

### Quantitative real-time PCR (qPCR)

qPCR was used to determine the relative abundance of bacteria. qPCR was carried out on the ABI 7500 fast real-time fluorescent quantitative PCR system (Thermo Fisher Scientific) using QIAampDNA Micro Kit (QIAGEN Sciences, Maryland USA) following the manufacturer’s instructions. The following sequences of primers we used were as previously described [32, 33]: *F. n*, forward 5′-CACAAGCTGACGCTGCTAGA-3′, reverse 5′-TTACCAGCTCTTAAAGCTTG-3′(232 bp); *F. p*, forward 5′-CCATGAATTGCCTTCAAAACTGTT-3′, reverse 5′-GAGCCTCAGCGTCAGTTGGT-3′ (141 bp). The relative abundances of *F. n* and *F. p* were calculated in reference to universal 16S rDNA, determined by qPCR using the following primers according to Caporaso et al. [34]: forward 5′-GTGCCAGCMGCCGCGGTAA-3′, reverse 5′-GGACTACHVGGGTWTCTAAT-3′ (291 bp). All primers were synthesized Sangon Biotech (Shanghai, China) Co., Ltd. Each sample was analyzed in triplicate in a single batch, and the average of the cycle threshold (Ct) values was calculated for the following analysis. The abundance was calculated as a relative unit normalized to the universal 16S rDNA of the same sample, using the 2^−ΔCt^ method (ΔCt = mean Ct _the target bacterial gene_ − mean Ct _16S rDNA_) [35, 36]. The Ct value is inversely associated with the amount of the target bacterial DNA, while the −ΔCt value is directly proportional to that; therefore, the higher the −ΔCt value, the greater the amount of the target bacterium was in the pharyngeal swab samples.

### Library construction and sequencing

Pharyngeal swab samples of 10 COVID-19 patients were randomly selected for metagenomic sequencing. The extracted RNA was quantified using a Qubit RNA High-Sensitivity kit (Invitrogen, USA) before library construction and sequencing. The library preparation was performed by KAPA Stranded RNA-Seq Library Preparation Kit (Kapa Biosystems, USA) following the manufacturer’s operational manual. Specifically, 10 μL total RNA was used as input and was fragmented by heating (94 °C, 8 min) into 150 ~ 200 nt fragments. The first-strand cDNA was synthesized in the presence of specific chemicals to ensure that only RNA was used as templates. Double strand cDNA was purified with Agencourt AMPure XP beads (Beckman Coulter, USA) after the reaction DNA library was constructed through end-repair, dA-tailing, adaptor-ligation, and 15 cycles PCR amplification. Subsequently, the resulting libraries were denatured, neutralized, and subject to 150 bp pair-end sequencing on an Illumina NovaSeq platform (Illumina, USA) by Chengdu HitGen Pharmaceuticals Inc., China. For each sample, 100 million reads were assigned and the Q30 of all runs ranged from 88 to 91%.

Raw reads were preprocessed using the metaWRAP-Read_qc module [37] with TrimGalore (https://www.bioinformatics.babraham.ac.uk/projects/trim_galore/) and BMTagger (ftp://ftp.ncbi.nlm.nih.gov/pub/agarwala/bmtagger/) enabled to remove adapters and host sequences, respectively. Sequences aligned to the SILVA rRNA gene database (https://academic.oup.com/nar/article/41/D1/D590/1069277/) were filtered from the processed datasets using Bowtie2 [38] before the de novo assembly (via MetaWRAP assembly module). Only contigs longer than 500 bp were kept for downstream analysis. Kraken2 [39] was used for taxonomic classification on the contigs, followed by Bracken [40] for the calculation of the species abundance.

### Sequence data process

The sequence data were processed through the following steps: a) The bacterial operational taxonomy units (OTUs) were then compared among samples by mothur software [41]. b) The relative abundances of bacterial taxa at the species level were calculated. The top 20 bacteria species were displayed.

### Statistical analysis

All continuous variables were presented as mean ± SD and categorical variables as percentage. All statistical analyses were carried out using the SPSS 26.0 software and GraphPad Prism 8.0 software. Normally distributed data were compared by *t*-test or one-way ANOVA, and non-normally distributed data were analyzed by Mann–Whitney *U* test. Logistic regression was used to screen and verify risk factors for SARS-CoV-2 infection. Pearson correlation analysis was used to analyze the correlation between the relative abundance of the other bacteria and SARS-CoV-2 or *F. n*. Biomarker performance was analyzed by calculating the area under the receiver operating characteristic (ROC) curve (AUC). Significant differences were considered at *P* < 0.05.

## Results

### Characteristics of the participants

For the healthy individuals who contributed pharyngeal swab samples, the median age was 43.5 years (interquartile range [IQR]: 31.5 ~ 47.5), with a male:female sex ratio of 1; the healthy fecal donors’ median age was 31.0 years (IQR: 28.0 ~ 44.0), with a male:female sex ratio of 0.8. Moderate illness accounted for the largest proportion of all the enrolled cases (66.67% ~ 70.43%), which was consistent with actually observed tendency [42, 43]. Asymptomatic infection, mild and severe ilness were less than 5% each. Data including age, sex, disease severity, the date of symptom onset (the day when the symptom was noticed), the sampling day and sampling intervals of the confirmed patients were registered (Table 1, Supplementary Tables 1–2). Indeed, retrospective cohort studies indicate that clinical course provides an objective basis for the egregation of patients into groups, and 7 days is an time node of great clinical importance [5, 43, 44]. Although we intended to group the samples by the sampling intervals (intervals from symptom onset to the sampling time) as ≤7 d, 7–14 d and > 14 d, the numbers of pharyngeal swab samples in the latter two groups were too small (*n* = 3 and 1, respectively) for a proper statistical analysis. Therefore, the latter two groups were combined and subsequently analyzed as a single group.

**Table 1:** Demographics and baseline characteristics of COVID-19 patients

	Pharyngeal swab samples (***n*** = 28)	Fecal samples (***n*** = 36)
**Age (yrs) - medium (IQR)**	36.0 (28 ~ 49.5)	46.5 (34.3 ~ 56.0)
**Age groups – No. (%)**		
≤ 30 yrs	11 (39.29)	8 (22.22)
31 ~ 50 yrs	10 (35.71)	15 (41.67)
>50 yrs	7 (25.00)	13 (36.11)
**Sex – No. (%)**		
Male	20 (71.43)	18 (50)
Female	8 (28.67)	18 (50)
**Severity of illness – No. (%)**		
Asymptomatic infection	4 (14.29)	3 (8.33)
Mild illness	4 (14.29)	4 (11.11)
Moderate illness	20 (71.43)	24 (66.67)
Severe illness	0	5 (13.89)
**Sampling intervals** ^**a**^ **–No. (%)**		
**≤ 7 d**	23 (85.2)	9 (26.5)
**> 7 d**	4 (14.8)	25 (73.5)
**SARS-CoV-2 RNA detection (%)**		
Positive (+)	28 (100)	26 (72.22)
Negative (−)	0 (0)	10 (27.78)

*Abbreviations*: *COVID-19* coronavirus disease 2019, *IQR* interquartile range

^a^ The analysis was unavailable in one of the pharyngeal swab samples and two of the fecal samples due to missing data on the onset time or sampling time

### Changes of the pharyngeal *F. n* in COVID-19 patients

**Fig. 1:**
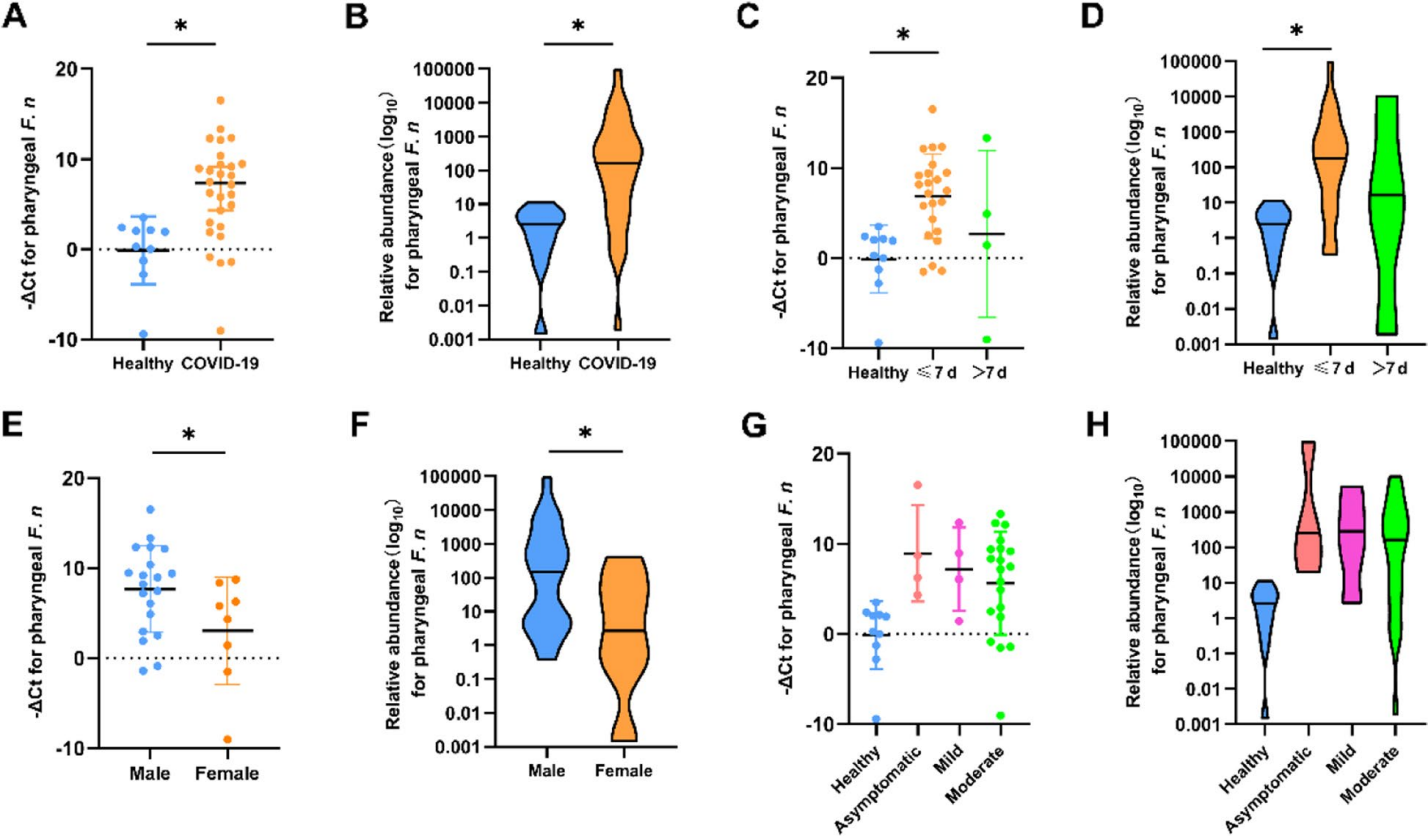
Quantitative detection of pharyngeal *F. n* in COVID-19 patients and healthy controls. The −ΔCt values and the relative abundance of pharyngeal *F. n* in the confirmed cases were compared with those in healthy controls (**A**, **B**), and were compared according to the sampling intervals (**C**, **D**), sex (**E**, **F**), and severity of illness (**G**, **H**). *P < 0.05

The −ΔCt values of the COVID-19 patients ranged from −9.03 to 16.52, and those of the healthy ranged from −2.78 to 3.50. As shown in Fig. 1A, the −ΔCt value of the confirmed cases (6.33 ± 5.47) was markedly higher than that of healthy controls (− 0.12 ± 3.77) (*P* < 0.05), which means that the relative abundance of pharyngeal *F. n* in COVID-19 patients was significantly higher than that in healthy controls (Fig. 1B). To analyze the impact of sampling intervals on the relative abundance of pharyngeal *F. n*, the patients were divided into two groups (≤ 7 d and > 7 d) according to the time interval from symptom onset to the sampling time. It was shown that within 7 days after the onset, the average − ΔCt value of COVID-19 patients was higher than that of healthy controls (*P* < 0.05). The pharyngeal *F. n* increased in the first week of illness (Fig. 1C, D). Interestingly, sex difference was found in the pharyngeal *F. n* of COVID-19 patients, and the average − ΔCt value of *F. n* in male patients was 10 times more than that in females (*P* < 0.05) (Fig. 1E, F). Furthermore, to determine the changes of pharyngeal *F. n* in patients with different severity of illness, we compared the −ΔCt level (Fig. 1G) and relative abundance (Fig. 1H) in those with asymptomatic (*n* = 4), mild (*n* = 3) and moderate illness (*n* = 20), and healthy controls (*n* = 10). It revealed that overall comparisons showed significant differences, while subsequent pairwise comparisons showed no significant differences (*P* >  0.05). In addition, the −ΔCt values of confirmed cases from different age groups (≤ 30 yrs., 31 ~ 50 yrs., and > 50 yrs) showed no significant difference between these groups (*P* >  0.05).

### Relationship between pharyngeal *F. n* and risk of viral infection

In the non-adjusted model, increased abundance of pharyngeal *F. n* was associated with a higher risk of a positive SARS-CoV-2 test (odds ratio [OR] = 1.29, 95% confidence interval [CI]: 1.06 ~ 1.57, *P* < 0.05). In the adjusted model (adjusted age and sex), the association between −ΔCt value and SARS-CoV-2 positive risk had a similar trend, but with a slightly raised magnitude (adjusted OR = 1.32, 95% CI = 1.06 ~ 1.65, *P* < 0.05), indicating that when the −ΔCt value of pharyngeal *F. n* increased by 1 (the relative abundance of *F. n* increased by 2), the risk of SARS-CoV-2 infection increased by 1.32 times.

### The relative abundance of *F. n* and *F. p* in the feces of COVID-19 patients didn’t alter much

**Fig. 2:**
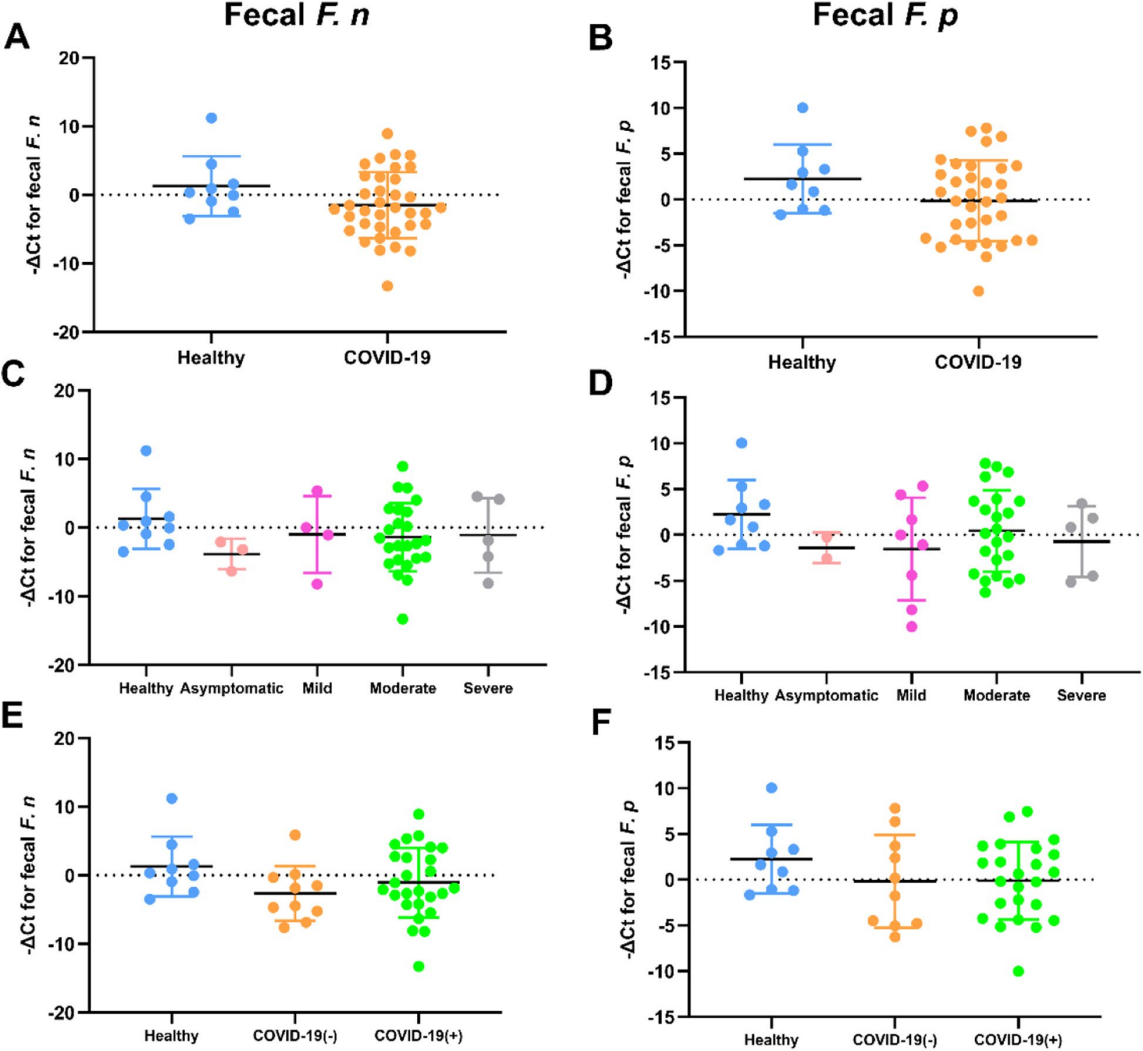
Quantitative detection of fecal *F. n* and *F. p* in COVID-19 patients and healthy controls. The −ΔCt values of fecal *F. n* and *F. p* in the confirmed cases were compared with healthy controls (**A**, **B**), and were compared according to the severity of illness (**C**, **D**). COVID-19 patients with either negative (−) or positive (+) SARS-CoV-2 RNA in the feces were compared with the healthy controls (**E**, **F**)

The relative abundance of fecal *F. n* and *F. p* in the healthy and confirmed cases was detected by qPCR. Though there was no significant difference (*P* > 0.05), the −ΔCt values of fecal *F. n* and *F. p* in the confirmed case group were slightly smaller than those of healthy controls (Fig. 2A, B). As shown in Fig. 2C and D, when comparing different severity with healthy controls, no significant difference in the −ΔCt values of both *F. n* and *F. p* was found (*P* > 0.05). Furthermore, we analyzed the 36 COVID-19 patients with SARS-CoV-2 RNA positive (*n* = 26) or negative (*n* = 10) in feces and found no significant difference in the −ΔCt values of fecal *F. n* and *F. p* among the two groups and healthy controls (*P* > 0.05) (Fig. 2E, F).

### Fecal *F. n* of COVID-19 patients decreased with sampling intervals prolongation

**Fig. 3:**
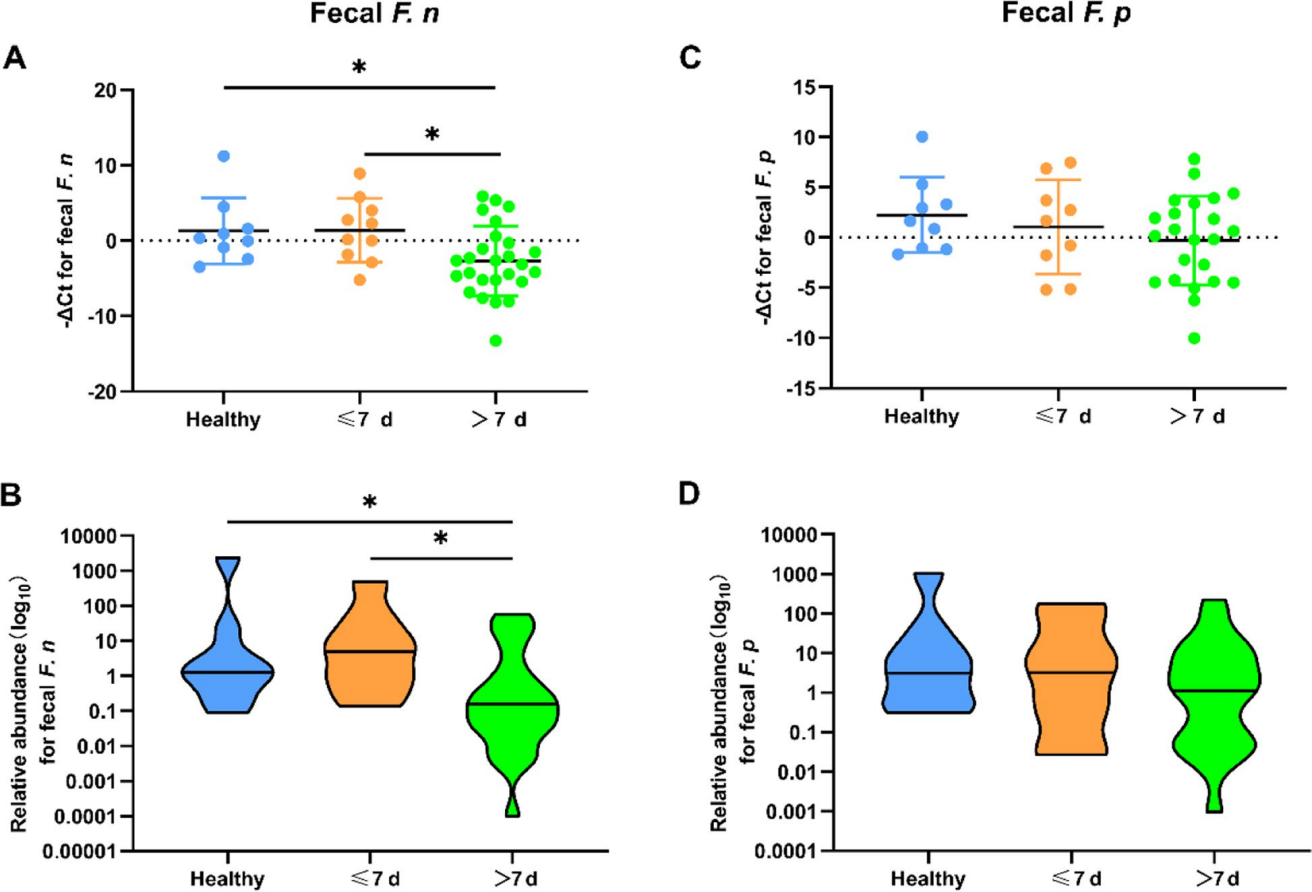
Quantitative detection of fecal *F. n* and *F. p* in different sampling intervals. The −ΔCt values and relative abundance of fecal *F. n* (**A**, **B**) and *F. p* (**C**, **D**) in different sampling intervals were analyzed. *P < 0.05

The confirmed cases were divided into two groups (≤ 7 d and > 7 d) according to the feces sampling intervals. It was shown that the fecal *F. n* of confirmed cases within 7 days after the onset was still at a healthy level (*P* > 0.05), but it was significantly lower after 7 days (*P* < 0.05) (Fig. 3A). In other words, in the cases with shorter feces sampling intervals (≤ 7 d), the relative abundance of fecal *F. n* had not changed significantly compared with the healthy individuals, but it decreased with the interval prolongation (Fig. 3B). Whereas, no significant difference was found in fecal *F. p* between the confirmed patients and the healthy controls (*P* > 0.05) (Fig. 3C and D).

### Neither fecal *F. n* nor *F. p* influenced the results of SARS-CoV-2 RNA test

Univariate logistic regression models were developed to explore the influence of fecal *F. n* or *F. p* on the results of SARS-CoV-2 RNA detection. First, we took the SARS-CoV-2 RNA test results in pharyngeal swabs of both the patients and the healthy indivduls as the dependent variables, and the −ΔCt values of fecal *F. n* or *F. p* as the independent variable for univariate logistic regression analysis. The results showed that neither fecal *F. n* nor *F. p* were independent risk factors of positive pharyngeal swabs (*P* > 0.05) (Table 2). Moreover, the influence of fecal *F. n* or *F. p* on the results of fecal SARS-CoV-2 RNA test in COVID-19 patients (i.e., negative or positive) was also analysed. However, it’s found that fecal *F. n* and *F. p* did not influence the results of fecal SARS-CoV-2 RNA testing (*P* > 0.05) (Table 3).

**Table 2:** Influence of fecal *F. n* and *F. p* on the results of pharyngeal swab SARS-CoV-2 RNA detection

Variables	OR	95%CI for OR	***P*** value
***F. n***	0.88	0.75 ~ 1.04	0.125
***F. p***	0.87	0.73 ~ 1.051	0.151

*Abbreviations: F. n Fusobacterium nucleatum*, *F. p Faecalibacterium prausnitzii*, *SARS-CoV-2* severe acute respiratory syndrome coronavirus 2, *OR* odds ratio, *CI* confidence interval

**Table 3:** Influence of fecal *F. n* and *F. p* on the results of fecal SARS-CoV-2 RNA detection

Variable	OR	95%CI for OR	***P*** value
***F. n***	1.08	0.92 ~ 1.26	0.386
***F. p***	1.00	0.85 ~ 1.19	0.965

*Abbreviations: F. n Fusobacterium nucleatum*, *F. p Faecalibacterium prausnitzii*, *SARS-CoV-2* severe acute respiratory syndrome coronavirus 2, *OR* odds ratio, *CI* confidence interval

### Identification of a microbial classifier for COVID-19 based on the microbial candidates

**Fig. 4:**
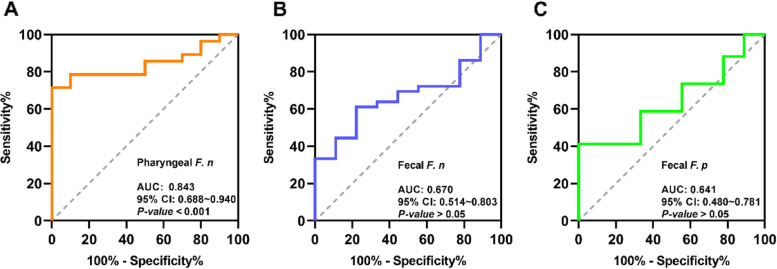
Diagnostic outcomes for *F. n* and *F. p* in the diagnosis of COVID-19. ROC curves for the diagnostic strength to identify COVID-19 from healthy controls with indicator of pharyngeal *F.n* (**A**), fecal *F. n* (**B**), and fecal *F. p* (**C**), respectively. AUC, area under the curve

A classifier to distinguish COVID-19 patients from the healthy controls based on the pharyngeal *F. n* was constructed and achieved an AUC of 0.843 (95% CI = 0.688 ~ 0.940, *P* < 0.001) (Fig. 4A). A value of 0.60 to 0.70 for AUC indicates that the predictive ability of the model is poor, and 0.8 to 0.9 is considered excellent [45]. Our data reveal that the pharyngeal *F.n* possessed a good accuracy in distinguishing COVID-19 patients from the healthy controls, the cutoff -ΔCT value for maximum sensitivity (71.43%) and specificity (100%) was 3.50. The sensitivity and specificity of various pharyngeal *F.n* cutoff -ΔCT values and 95% CI are shown in Table 4. However, the fecal *F. n* (Fig. 4B) and *F. p* (Fig. 4C) cannot be used for prediction because of their poor discrimination ability.

**Table 4:** Sensitivity and specificity according to various cutoff values for the -ΔCt of pharyngeal *F.n* in qPCR to predict COVID-19

-ΔCt cutoff	Sensitivity (95% CI)	Specificity (95% CI)
≥ −9.41	100 (87.7 ~ 100.0)	0 (0.0 ~ 30.8)
> − 9.41	100 (87.7 ~ 100.0)	10 (0.3 ~ 44.5)
> − 9.03	96.43 (81.7 ~ 99.9)	10 (0.3 ~ 44.5)
> − 2.78	96.43 (81.7 ~ 99.9)	20 (2.5 ~ 55.6)
> − 1.42	89.29 (71.8 ~ 97.7)	20 (2.5 ~ 55.6)
> − 1.27	89.29 (71.8 ~ 97.7)	30 (6.7 ~ 65.2)
> − 0.88	85.71 (67.3 ~ 96.0)	30 (6.7 ~ 65.2)
> 0.27	85.71 (67.3 ~ 96.0)	50 (18.7 ~ 81.3)
> 1.91	78.57 (59.0 ~ 91.7)	50 (18.7 ~ 81.3)
> 2.40	78.57 (59.0 ~ 91.7)	90 (55.5 ~ 99.7)
> 2.96	71.43 (51.3 ~ 86.8)	90 (55.5 ~ 99.7)
**> 3.50**	**71.43 (51.3 ~ 86.8)**	**100 (69.2 ~ 100.0)**
> 16.52	0 (0.0 ~ 12.3)	100 (69.2 ~ 100.0)

*Abbreviations*: *Ct* cycle threshold, *F. n Fusobacterium nucleatum*, *qPCR* quantitative real-time polymerase chain reaction, *COVID-19* coronavirus disease 2019, *CI* confidence interval

### Enrichment of opportunistic pathogens in the pharyngeal swabs of COVID-19 patients

**Fig. 5:**
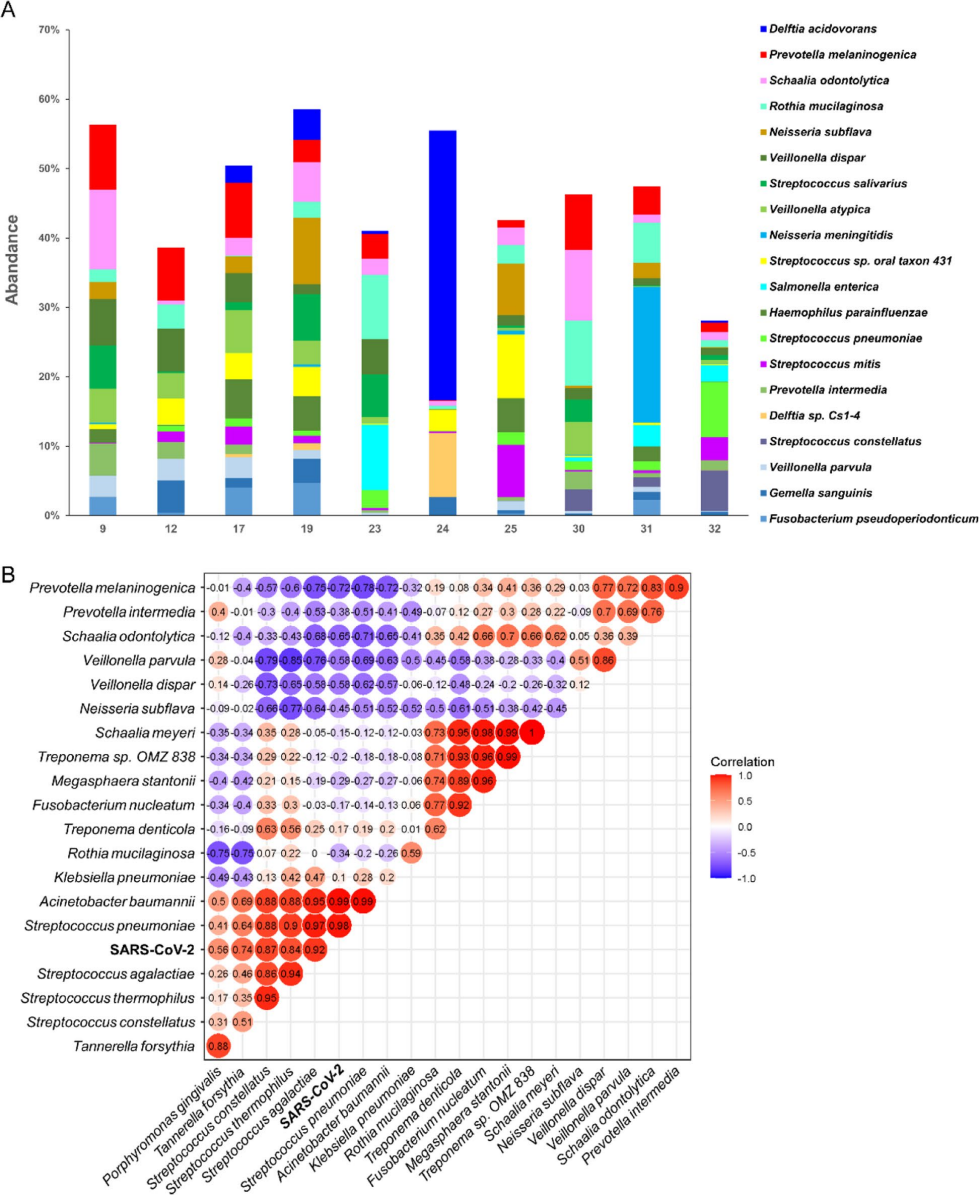
The pharyngeal microbiome analysis of patients with COVID-19. The top 20 pharyngeal microbial species composition in each pharyngeal sample (**A**). Correlation between pharyngeal bacteria and viral loads in COVID-19 patients (**B**)

The number of OTUs in 10 COVID-19 patients was 613 ± 266. And the relative abundance of SARS-CoV-2 was 0.077% (9^#^), 0.017% (12^#^), 0.008% (17^#^), 0.012% (19^#^), 0.002% (23^#^), 0.029% (25^#^), 0.021% (30^#^), 0.009% (31^#^), and 1.982% (32^#^), respectively. The pharyngeal microbiome of each COVID-19 patient was dominated by the top 20 species, accounting for 28.12 to 58.55% of the total richness (Fig. 5A). The predominant bacterial composition in the pharyngeal swabs of the confirmed patients included *Prevotella melaninogenica* (4.62% ± 3.17%), *Schaalia odontolytica* (3.85% ± 3.78%), *Rothia mucilaginosa* (3.62% ± 3.24%), *Neisseria subflava* (3.06% ± 3.33%), and *Veillonella dispar* (2.89% ± 2.29%). Interestingly, 7 out of 10 samples harbored a high abundance of *Delftia acidovorans* (6.65% ± 13.24%), especially for sample 24^#^, which showed the least amount of OTUs and the highest relative abundance of *Delftia acidovorans* (38.87%).

To explore the relationship of the viral loads with the common bacteria in the pharynx of COVID-19 patients, Pearson’s correlation analysis was performed. As shown in Fig. 5B, the viral loads in the pharyngeal swabs of COVID-19 patients showed a positive linear correlation with the abundance of *Streptococcus pneumoniae*, *Streptococcus thermophilus*, *Acinetobacter baumannii*, and *Streptococcus constellatus* (*P* < 0.05). Besides, the abundance of *F. n* correlated with *Treponema denticola* (*P* < 0.05).

## Discussion

Virus-bacteria interactions are complicated. Our study reveal that the pharyngeal *F. n* of COVID-19 patients was increased, which deserves special attention. The increased abundance of pharyngeal *F. n* may promote SARS-CoV-2 infection, for *F. n* can induce ACE2 expression and proinflammatory cytokine production and thus promote SARS-CoV-2 invasion and infection in oral, respiratory and intestinal epithelial cells [23]. Dysbiosis of microbiome in the oral cavity may impact distant microbiomes via the oral–lung or oral–gut axis [46]. Several studies reported that *F. n* was detected in the lungs of ICU patients [47, 48]. Increasing evidence has shown that *F. n* is closely related to the development of inflammatory bowel disease and even colorectal cancer [21, 22]. Hence, we can rationally infer that bacterial coinfection after translocation of *F. n* would aggravate COVID-19. SARS-CoV-2 infection of oral cavity would lead to local microbial dysbiosis [7], and thus make the oral cavity as a reservoir for pathogens. Since the abundance of pharyngeal *F. n* in COVID-19 patients increased, more focus should be given to preventing periodontal diseases and systemic diseases [49].

Epidemiological data have shown that the morbidity, severity and mortality rates of COVID-19 are higher in males than females [50]. Several possible factors such as the higher expression of ACE2 in male, and sex-based immunological differences driven by sex hormone and X chromosome may be involved [51]. Besides, studies have revealed significant sexual differences in oral hygiene worldwide [52–54]. Importantly, we found that the pharyngeal *F. n* of SARS-CoV-2-positive males was higher than that of females. Based on the above researches and our findings, we speculate that poor oral health may lead to higher morbidity and mortality of COVID-19 in men.

Retrospective cohort studies suggested that 1 week after onset of symptoms, patients with COVID-19 may deteriorate rapidly [5, 43, 44]. Notably, the highest SARS-CoV-2 load was detected in the upper respiratory tract at the time of symptom onset or in the first week of illness [55]. Interestingly, in our results, the pharyngeal *F. n* was higher than that of healthy controls in the first week of illness. We speculate that pharyngeal *F. n* may associated with the severity or viral load. Unfortunately, our sequencing data did not reveal the correlation. Further large-sample longitudinal studies are required to confirm this speculation. What’s more, our findings valuable information for the analysis of the pharyngeal and gut microbiota profiles of COVID-19 patients at different time points after SARS-CoV-2 infection. However, understanding whether *F. n* or *F. p* alters in the course of COVID-19 will require larger cohort studies, with a focus on longitudinal responses following initial infection.

A diverse gut microbiome promotes the host health, and commensal microbiota can inhibit the invasion and growth of pathogens [56]. Previous studies showed that COVID-19 patients demonstrated a depletion of *F. p* [25], and the abundance of *F. p* was inversely correlated with disease severity [26]. Our study, however, found that the abundance of fecal *F. p* in the infected individuals was not significantly different from those of the healthy, which may partially due to the distinct clinical classification and sampling time.

The dominant oral commensal bacteria in the pharyngeal swabs of the confirmed patients, like *Prevotella melaninogenica*, *Schaalia odontolytica*, *Rothia mucilaginosa*, *Neisseria subflava*, and *Veillonella dispar*, was consistent with what previous studies reported [57, 58]. Notably, *Prevotella melaninogenica* and *Rothia mucilaginosa* were found overrepresent in the lungs of COVID-19 patients [58, 59]. Additionally, functional enrichment analysis found that over-expressed Prevotella proteins were related to the aggravation of COVID-19 [60], suggesting that *Prevotella* might play an important role in the progression of COVID-19. *Rothia mucilaginosa* was reported to be associated with pneumonia in patients with chronic obstructive pulmonary disease (COPD) [61]. Unsurprisingly, opportunistic respiratory pathogens, including *Streptococcus pneumoniae, Streptococcus thermophilus, Acinetobacter baumannii,* and *Streptococcus constellatus*, were found to be positively correlated with viral loads, which in turn explains the respiratory disorders following viral infection. These results demonstrate that vigilance should be maintained when respiratory virus infection occurs, and attention should be paid to the secondary bacterial infection. We also found that the relative abundance *Delftia acidovorans* significantly increased in the pharyngeal swabs of SARS-CoV-2-infected individuals, and it was dominant (38.87%) in sample 24. As reported, *Delftia acidovorans* was found in tracheal lavage samples of COVID-19 patients admitted to intensive care units, with abundance of almost 6% [62]. It is usually nonpathogenic, yet catheter-related infection and pneumonia with lung cavity formation have been reported [63, 64]. Further studies are needed to explore the correlation between *Delftia acidovorans* and symptoms of COVID-19 such as coagulopathy and disseminated intravascular coagulation, and to illustrate the potential mechanism of this dominant species and the decrease of OTUs.

Moreover, our study suggested that the pharyngeal *F. n* could specifically identify patients with COVID-19 from the healthy controls, and thus it may be a promising candidate indicator (AUC = 0.843) for SARS-CoV-2 infection. Similar to our study, a recent study [7] analysed tongue-coating samples and found that several oral microbial markers (like TM7, *Haemophilus*, *Actinomyces*, *Prevotella*, *Oribacterium* and *Fusobacterium*) could serve as an auxiliary tool for the non-invasive diagnosis of COVID-19. Previous investigations have demonstrated the diagnostic value of oral microbiome for colorectal cancer [27], rheumatoid arthritis [65] and even viral diseases [12]. Meanwhile, we first proposed that the pharyngeal *F. n* could be a reliable indicator for prospective epidemiological monitoring and large-scale screening of COVID-19. We concede that large prospective cohort studies, which account for possible confounders such as age, sex, systemic diseases and oral microbiota composition, are needed to verify the diagnostic efficacy.

## Conclusions

In summary, the present study provide potentially significant clinical findings. We reported that the pharyngeal *F. n* was significantly increased in COVID-19 patients. Thus, more attention from the public and medical communities is warranted for the alterations in the oral microbiome of COVID-19. Effective oral hygiene measures and promotions are necessary to avoid the coinfection by oral microorganisms and the following disease aggravation.

## Supplementary Information

**Additional file 1.**

## Data Availability

The datasets generated and analysed during the current study are available in the 2019 Novel Coronavirus Resource (2019nCoVR) (https://bigd.big.ac.cn/ncov/).

## Abbreviations

COVID-19: Coronavirus disease 19
SARS-CoV-2: Severe acute respiratory syndrome coronavirus 2
ACE2: Angiotensin-converting enzyme 2
TMPRSS2: Transmembrane serine protease 2
*F. n*: *Fusobacterium nucleatum*
*F. p*: *Faecalibacterium prausnitzii*
qPCR: Quantitative real-time polymerase chain reaction
mNGS: Metagenomic next-generation sequencing
Ct: Cycle threshold
OTUs: Operational taxonomy units
ROC: Receiver operating characteristic
AUC: Area under the ROC curve
IQR: Interquartile range
CI: Confidence interval
OR: Odds ratio
